# Therapeutic Effects and Mechanisms of Fucoidan on Dyslipidemia in Obese Mice

**DOI:** 10.1002/fsn3.72159

**Published:** 2026-09-15

**Authors:** Lizhi Peng, Ruiming Li, Ruting Zheng, Ziqiong Liao, Daohuang Luo, Yuting Zeng, Jiaqi Liu, Qiaoling Kong, Jicai Lai

**Affiliations:** ^1^ Department of Pharmacy, Shenzhen Key Laboratory of Chinese Medicine Active Substance Screening and Translational Research The Seventh Affiliated Hospital of Sun Yat‐Sen University Shenzhen China; ^2^ State Key Laboratory of Quality Research in Chinese Medicine, School of Pharmacy Macau University of Science and Technology Macau China; ^3^ School of Pharmacy Guangdong Food and Drug Vocational College Guangzhou Guangdong Province China; ^4^ Department of Traditional Chinese Medicine The Seventh Affiliated Hospital of Sun Yat‐Sen University Shenzhen China

**Keywords:** dyslipidemia, fucoidan, liver, Pla2g2f, TNFα

## Abstract

Dyslipidemia, characterized by abnormal levels of serum lipids such as cholesterol and triglycerides, is a significant risk factor for metabolic disorders. Fucoidan, a marine sulfated polysaccharide, has shown anti‐inflammatory and lipid‐modulating properties, but its mechanism of action in high‐fat diet (HFD)‐induced dyslipidemia remains unclear. In this study, treatment with fucoidan (50 mg/kg/day) did not alter HFD‐driven weight gain or food intake, but significantly decreased serum total cholesterol (TC). Although serum high‐density lipoprotein cholesterol (HDL‐C) was also decreased, this change may reflect normalization of HFD‐induced cholesterol homeostasis in mice. Moreover, fucoidan treatment reduced hepatic triglyceride and TC, accompanied by decreasing lipid droplets in the liver, indicating fucoidan is critical for improving HFD‐induced hepatic lipid accumulation. RNA‐seq analysis revealed that fucoidan reverses HFD‐induced downregulation of Defa24, Slc34a1, and Nnt, and may contribute to maintain hepatic metabolic homeostasis. Network pharmacology identified TNFα as a candidate key target, and molecular docking suggested a potential interaction between fucoidan and TNFα. Furthermore, fucoidan suppressed HFD‐driven upregulation of TNFα and its downstream target Pla2g2f in vivo and attenuated TNFα‐induced Pla2g2f expression in RAW264.7 cells. Collectively, these findings suggest that the protective effects of fucoidan against HFD‐induced dyslipidemia are associated with regulation of cholesterol metabolism and suppression of TNFα–Pla2g2f‐associated inflammatory signaling, supporting its potential application in dyslipidemia management.

## Graphical abstract

In the present study, fucoidan (50 mg/kg/day) did not alter HFD‐driven weight gain or food intake, but significantly decreased serum total cholesterol (TC). Fucoidan reduced hepatic triglyceride, TC, and lipid droplets, improving hepatic lipid accumulation. RNA‐seq revealed that fucoidan reverses HFD‐induced downregulation of Defa24, Slc34a1, and Nnt. Network pharmacology and molecular docking identified TNFα as a candidate key target. Furthermore, fucoidan suppressed HFD‐driven upregulation of TNFα and its downstream gene Pla2g2f in vivo, and attenuated TNFα‐induced Pla2g2f expression in RAW264.7 cells. These protective effects against HFD‐induced dyslipidemia are associated with regulation of cholesterol metabolism and suppression of TNFα–Pla2g2f‐associated inflammatory signaling.

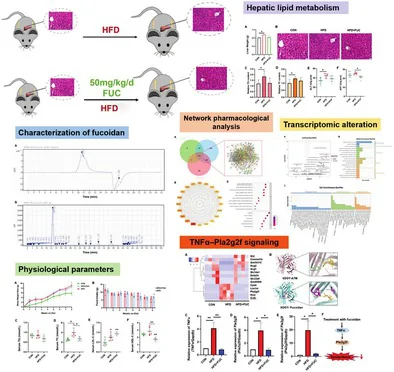

## 1 Introduction

Dyslipidemia, characterized by abnormal levels of serum lipids such as cholesterol and triglycerides, is a significant risk factor for metabolic disorders including obesity, atherosclerosis, and non‐alcoholic fatty liver disease (NAFLD) (Nussbaumerova and Rosolova 2023; Zeljkovic et al. 2024). The increasing prevalence of obesity has exacerbated the incidence of dyslipidemia, posing substantial challenges to human health (Vekic et al. 2023). However, statins and fibrates are commonly used to manage dyslipidemia; their long‐term application is often accompanied by adverse effects such as liver toxicity and gastrointestinal disturbances (Modica et al. 2024; Chapman et al. 2010). These limitations have intensified interest in identifying safe and effective therapeutics for the prevention and treatment of dyslipidemia (Chehade et al. 2013).

Fucoidan, a sulfated polysaccharide derived from brown seaweeds like Laminaria japonica, is known for its potential promising applications (Fitton et al. 2019). Multiple studies have shown that fucoidan has beneficial pharmacological effects, including anti‐cancer, anti‐inflammatory, anti‐obesity, and anti‐diabetes effects (Atashrazm et al. 2015; Boo et al. 2013; Yokota et al. 2016; Zuo et al. 2022; Zheng et al. 2018). Previous studies have shown that fucoidan can significantly reduce total cholesterol, triglycerides, and LDL‐C in mice, and effectively prevent the formation of hypercholesterolemia in mice (Lee, Jayawardena, et al. 2022; Yokota et al. 2016). These findings highlighted fucoidan's potential in regulating lipid metabolism pathways, including the inhibition of lipogenesis and the promotion of lipid oxidation. In addition, fucoidan exerts its hypolipidemic effects through multiple pathways (Chu et al. 2025; Kim et al. 2014; Lin et al. 2025). It has been shown to inhibit the transcription factor‐kappa B (NF‐κB) pathway, leading to decreased expression of pro‐inflammatory cytokines such as tumor necrosis factor α (TNFα) and interleukin 6 (IL‐6), which are implicated in the progression of metabolic disorders (Chu et al. 2025; Lin et al. 2025).

Excess fat accumulation in the hepatocytes to promote lipid droplets formation may be involved in the pathophysiology of metabolic disorders, which is closely associated with dyslipidemia (Carr and Ahima 2016; Katsiki et al. 2016). Notably, fucoidan can significantly improve hepatic steatosis and liver injury (Kim et al. 2014; Zheng et al. 2018; Li et al. 2025). Our previous study showed that 500 mg/kg fucoidan improved obesity‐induced metabolic dysfunction by enhancing thermogenesis and promoting mitophagy through cAMP‐PKA‐PGC1α pathway, and thus protecting against obesity (Peng et al. 2025). According to these promising findings, the precise molecular targets and pathways through which fucoidan exerts its lipid metabolic regulatory effects require further study. Therefore, this study aims to reveal the mechanisms by which fucoidan ameliorates dyslipidemia induced by a high‐fat diet (HFD) in mice. By integrating network pharmacology, transcriptomic analyses, and molecular docking studies, we identified key targets and pathways altered by fucoidan treatment, contributing to provide a comprehensive understanding of its therapeutic potential in dyslipidemia.

## 2 Materials and Methods

### 2.1 Animal Experiment

The animal experiment protocols were authorized by the Animal Ethics Committee of Peking University Shenzhen Graduate School (AP0044026). Six‐week‐old male C57BL/6 mice were acclimatized for 1 week before the experiment. The mice were randomly divided into three groups (*n* = 5), including a normal‐diet control group (CON), a high‐fat diet (HFD) group (60% kcal from fat), and a high‐fat diet group with daily oral administration of 50 mg/kg fucoidan (HFD + FUC). The dose of fucoidan was selected based on previous studies demonstrating its efficacy in HFD‐induced dyslipidemia (Huang et al. 2021). Notably, fucoidan from Laminaria japonica was purchased from Shanghai Yuanye Bio‐Technology Co. Ltd. (Shanghai, China). According to the product information, the purity of the fucoidan was specified as 85% (bioreagent grade), and the detected methods referred to a previous report (Hu et al. 2026). Body weight and food intake were recorded daily over the course of 8 weeks. At the end of the experiment, all mice were euthanized by cervical dislocation. Blood samples were collected, liver weights were measured, and interscapular white adipose tissue (iWAT), epididymal white adipose tissue (eWAT), and brown adipose tissue (BAT) were collected for further study.

### 2.2 Hematoxylin–Eosin (H&E) Histomorphology Examination

Tissue samples were fixed in 4% formaldehyde for 24 h, embedded in paraffin, and sectioned into 5 μm thick slices. The sections were stained with H&E according to a standard protocol (Beyotime Biotechnology, Shanghai, China), including hematoxylin staining (5 min), differentiation in 1% acid alcohol (3 s), bluing in lithium carbonate solution (1 min), and eosin staining (30 s). Histological images were captured using an optical microscope at ×200 magnification.

### 2.3 Determination of Serum Biochemical Indexes

Serum was obtained by centrifugation from blood samples collected from mouse eyeballs. Serum biochemical indexes of mice were determined by an automatic biochemical analyzer. Serum levels of total cholesterol (TC), triglyceride (TG), alanine aminotransferase (ALT), and aspartate aminotransferase (AST) were measured according to the manufacturer's protocols (Nanjing Jiancheng Bioengineering Institute, Nanjing, China).

### 2.4 Transcriptomics Analysis

Liver tissue was utilized for transcriptomics analysis. For transcriptome sequencing, each group contained four biological samples (*n* = 4). The RNA‐sequencing (RNA‐seq) was conducted by Lianchuan Biotech (LC‐Bio Technology Co. Ltd., Hangzhou, China), as previous report (Peng et al. 2025). Differentially expressed genes were identified using the DESeq2 package (v1.24.0) in R, with genes showing an adjusted *p*‐value < 0.05 and an absolute log2 fold change (|log2FC|) > 1 considered significant (Table S1). To assess the overall distribution of gene expression profiles across the experimental groups, principal component analysis (PCA) and hierarchical clustering were performed. Volcano plots were generated to illustrate the overall distribution of differentially expressed genes, and a heatmap‐based cluster analysis was conducted to visually represent the similarity of gene expression profiles of all samples.

### 2.5 Enrichment Analysis, Protein–Protein Interaction (PPI), and Network Construction

Gene Ontology (GO) and Kyoto Encyclopedia of Genes and Genomes (KEGG) pathway enrichment analyses were performed using the R software (v3.6.1). KEGG pathways were identified to determine the biological functions and signaling pathways associated with the differentially expressed genes. “GOplot” is used to visually create the bubble map, including biological processes (BP), cell genesis (CC), and molecular functions (MF).

### 2.6 Molecular Docking

The three‐dimensional structure of TNF‐α was obtained from the Protein Data Bank (PDB ID: 6OOY), and the optimized fucoidan structure was converted to the appropriate file format for docking. Molecular docking was conducted using AutoDock Vina. Docking parameters were set to default values unless otherwise specified, and multiple docking runs were performed to ensure reproducibility of binding poses. The resulting docking poses were ranked by binding energy, and the top‐scoring conformation was selected for further analysis. The interactions between fucoidan and key residues of TNF‐α were visualized using PyMOL, and hydrogen bond interactions and hydrophobic contacts were identified to elucidate the binding mechanism.

### 2.7 Cell Culture and TNFα Stimulation Assay

RAW264.7 cells were obtained from the Cell Bank of the Chinese Academy of Sciences (Shanghai, China), and cultured in Dulbecco's Modified Eagle Medium (DMEM) supplemented with 10% fetal bovine serum (FBS) and 1% penicillin–streptomycin at 37°C in a humidified atmosphere containing 5% CO_2_. To evaluate the regulatory effect of fucoidan on TNFα‐induced Pla2g2f expression, cells were divided into three groups: Control, TNFα (10 ng/mL), and TNFα (10 ng/mL) + FUC (50 μg/mL). After 12 h of incubation, cells were harvested for total RNA extraction.

### 2.8 Real‐Time Reverse Transcriptase‐Polymerase Chain Reaction (RT‐qPCR) Investigation

Liver tissue was isolated using Trizol reagent (Invitro‐gen, USA) RNA and cDNA were synthesized by RevertAid reverse transcription kit (Thermo Scientific, USA) as previous study (Liu et al. 2021). The subsequent RT‐qPCR step was performed using the SYBR Green mix kit (Thermo Scientific, USA). The relative expression level was calculated using the 2^−ΔΔCt^ method, and the expression level of GAPDH mRNA was normalized as internal control. RT‐qPCR analyses were performed using three independent biological replicates, with each sample analyzed in technical triplicate.

### 2.9 Statistical Analysis

All quantitative data are expressed as mean ± SEM. Statistical analyses were performed using GraphPad Prism 8.0.2. Comparisons among multiple groups were analyzed using one‐way analysis of variance (ANOVA), followed by Tukey's multiple‐comparison test. A value of *p* < 0.05 was considered statistically significant. A value of *p < 0.05* was considered statistically significant.

## 3 Results

### 3.1 Fucoidan Ameliorates Dyslipidemia Induced by HFD in Mice

**FIGURE 1:**
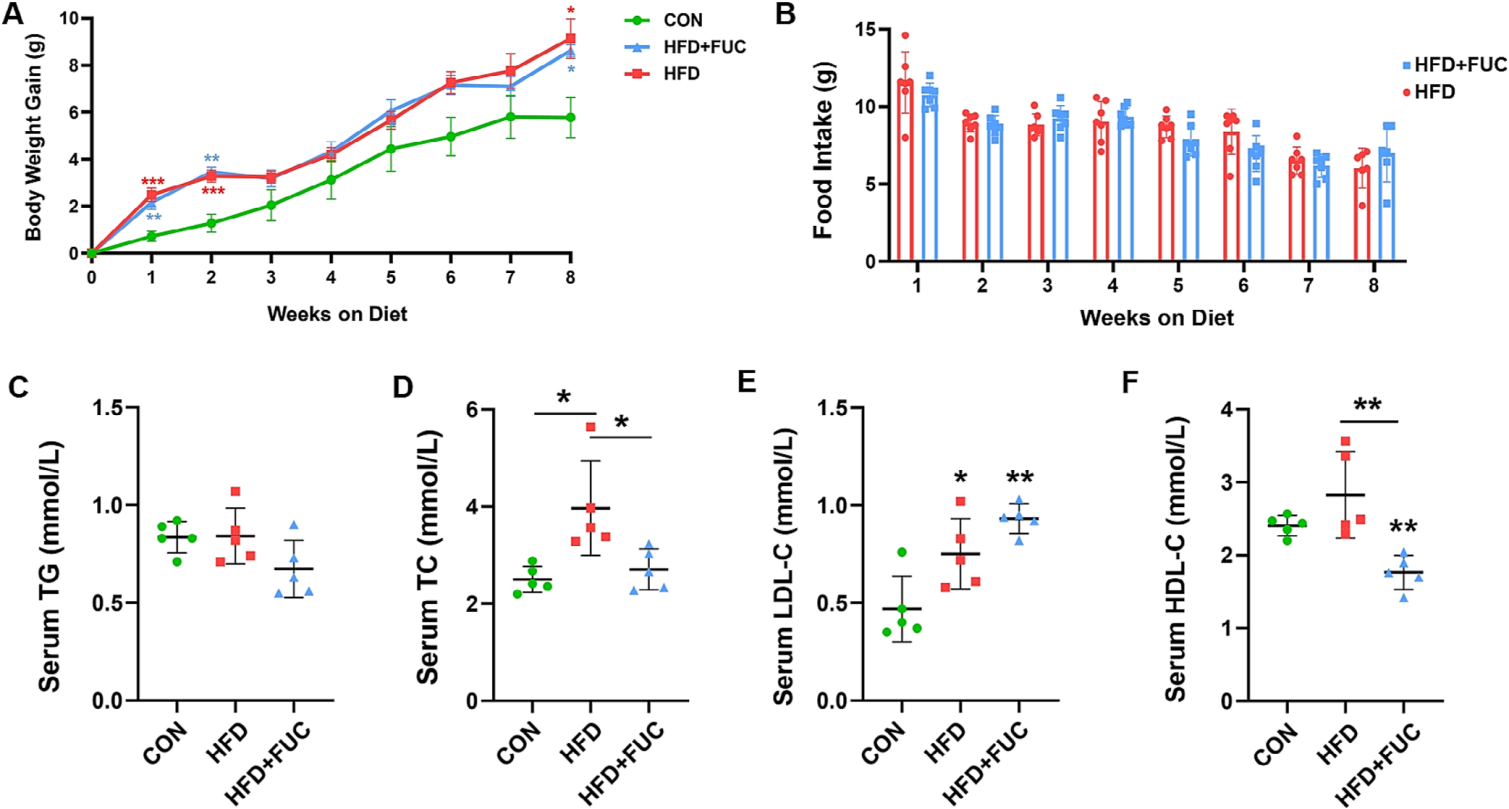
Low‐dose of fucoidan improves high‐fat diet‐induced dyslipidemia. (A, B) Body weight (A) and food intake (B) of mice fed with normal diet (Ctrl), high‐fat diet (HFD), high‐fat diet treated with 50 mg/kg/d fucoidan (HFD + FUC). (C) The serum triglyceride (TG) level of mice. (D) The total cholesterol (TC) of mice. (E, F) The serum LDL‐C (E) and HDL‐C (F) of mice. Results presented as means ± SEM of five independent experiments (*n* = 5). *p < 0.05. **p < 0.01.

HPGPC analysis showed that the fucoidan preparation exhibited a predominant and relatively symmetrical chromatographic peak, indicating a relatively homogeneous molecular weight distribution, showing an average molecular weight of 270.4 kDa (Figure 1A). The total sugar and sulfate contents were 86.3% ± 1.4% and 31.8% ± 0.6%, respectively (Table S2). Monosaccharide composition analysis by HPLC revealed that fucoidan was mainly composed of L‐fucose (48.88%) and D‐galactose (18.70%), together with smaller amounts of L‐rhamnose (10.48%), D‐xylose (9.52%), D‐mannose (7.88%), and D‐glucosamine (4.54%), indicating that the fucoidan preparation was a fucose‐rich sulfated polysaccharide (Figure 1B, Table S2). To elevate the anti‐obesity effects of fucoidan treatment (FUC), we established an animal model of obesity induced by a high‐fat diet (HFD). We found HFD significantly altered body weight gain in the first week, while 50 mg/kg fucoidan treatment did not alter HFD‐induced obesity (Figure 1A). Moreover, the fucoidan treatment did not alter the food intake when compared with HFD (Figure 1B). Although the level of serum triglycerides (TG) was similar in each group, increased levels of total cholesterol (TC) and low‐density lipoprotein cholesterol (LDL‐C) were observed in the HFD group compared with the control (CON) group (Figure 1C–F). Fucoidan treatment decreased TC (Figure 1D), indicating improvements in HFD‐induced dyslipidemia by lowering cholesterol metabolism.

### 3.2 Fucoidan Treatment Ameliorated HFD‐Induced Hepatic Lipid Accumulation

**FIGURE 2:**
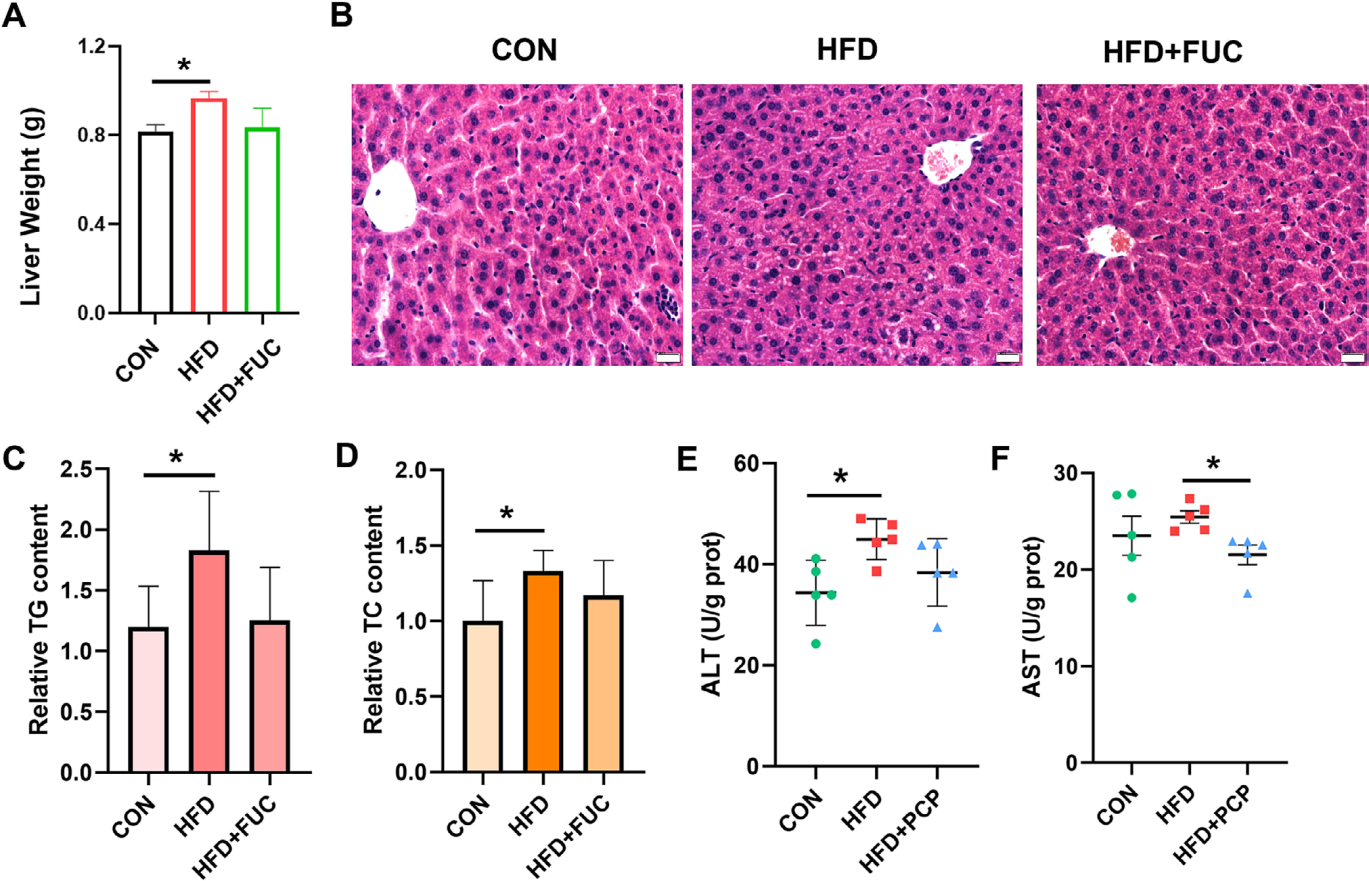
Fucoidan treatment improves lipid accumulation in liver induced by high‐fat diet. (A) The liver weight of mice fed with normal diet (Ctrl), high‐fat diet (HFD), high fat diet treated with 50 mg/kg/d fucoidan (HFD + FUC). (B) The representative H&E staining of liver tissue. Scale bar, 50 μm. (C, D) Relative TG (C) and TC (D) contents of liver tissue. (E, F) ALT (E) and AST (F) levels of liver tissue. Data are expressed as mean ± SEM (*n* = 5). *p < 0.05. **p < 0.01.

After eight weeks, HFD mice exhibited a significant increase the weights of liver, inguinal white (iWAT), epididymal white (eWAT), and brown adipose tissue (BAT) (Figure 2A, Figure S2A–C). H&E staining result showed that HFD led to an increased lipid droplet in liver, which could be reversed by fucoidan treatment (Figure 2B). These histopathological alterations reflect fucoidan treatment can reverse the severe lipid accumulation and inflammatory injury induced by HFD. Furthermore, fucoidan treatment reversed HFD induced increasing contents of TG and TC in liver (Figure 2C,D). In addition, fucoidan significantly reduced the levels of alanine aminotransferase (ALT) and aspartate aminotransferase (AST) in liver (Figure 2E,F), indicating that fucoidan alleviated liver injury and improved liver function.

### 3.3 Identification and Functional Characterization of the Anti‐Obesity and Dyslipidemia Targets of Fucoidan

**FIGURE 3:**
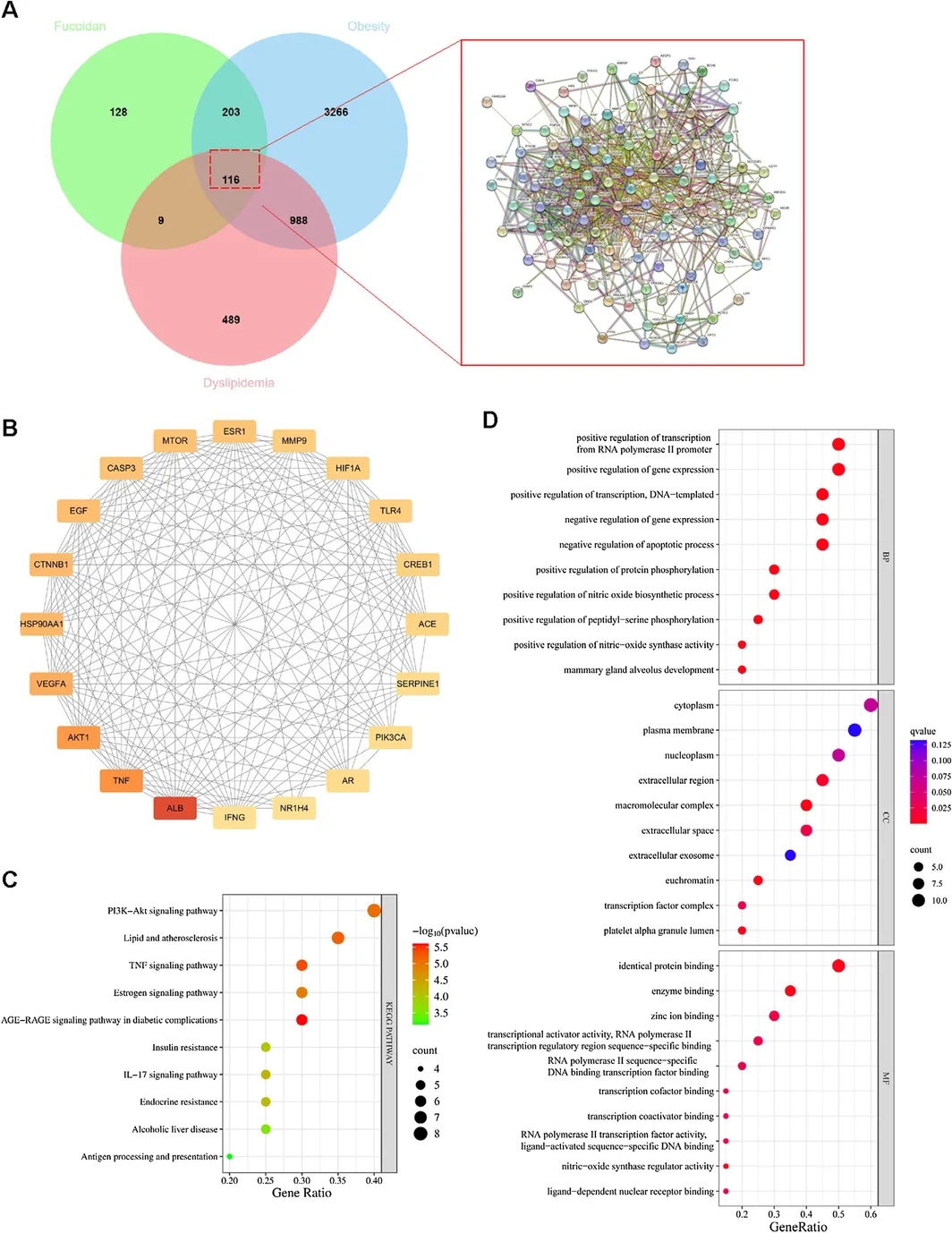
Identification and functional characterization of fucoidan's targets against obesity and dyslipidemia. (A) A Venn diagram showed the number of shared fucoidan, obesity, and dyslipidemia related genes (left panel). The protein–protein interaction of the ten core target genes was analyzed using STRING (right panel). (B) PPI network analysis identified 20 core target genes of fucoidan's targets against obesity and dyslipidemia. (C) The target genes were analyzed by statistical analysis of the KEGG pathway enrichment scatter plot (top 10). (D) GO enrichment analysis of target genes, which included the biological process (BP), cell component (CC), and molecular function (MF) categories. The size of the bubble represented the number of involved genes, and the color of the bubble represented the significance of the terms.

We performed a comprehensive network pharmacological analysis to identify representative genes associated with fucoidan, obesity, and dyslipidemia, resulting in a total of 456 genes related to fucoidan treatment, 4573 pathogenic genes related to obesity, and 1602 genes related to dyslipidemia (Figure 3A). As shown in Venn diagram data, fucoidan has 116 shared genes among targets against obesity and dyslipidemia. Twenty genes were identified as core targets, including ALB (albumin gene), TNF (tumor necrosis factor), AKT1 (serine/threonine kinase 1), VEGFA (vascular endothelial growth factor A) (Figure 3B). According to KEGG pathway enrichment analysis, core genes were enriched in phosphatidylinositol 3‐kinase (PI3K)/AKT pathway ((PI3K/AKT), TNF, and IL‐17) signaling pathways (Figure 3C). Go pathway result showed that fucoidan may negative regulate apoptotic process, and positive regulate nitric oxide biosynthetic pathway and nitric‐oxide synthase activity, suggesting fucoidan treatment ameliorates dyslipidemia through regulation of inflammatory and immune pathways.

### 3.4 RNA‐Seq Analysis of Differentially Expressed Genes (DEGs) of Fucoidan Treatment Ameliorates Dyslipidemia Induced by HFD in Liver

**FIGURE 4:**
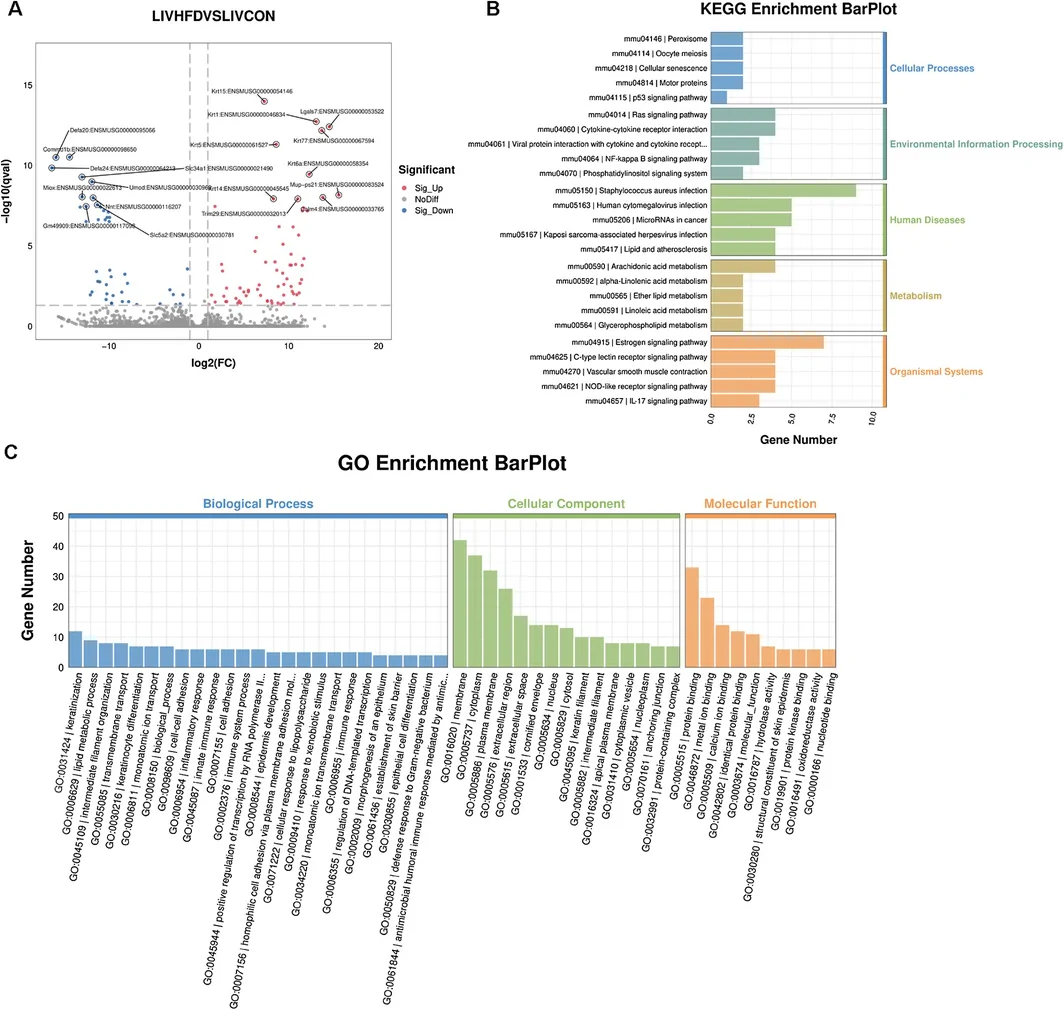
High fat diet alters gene expression levels in liver of mice. (A) Volcano plot of differentially expressed genes in HFD group versus Ctrl group. (B) The differentially expressed genes enriched in KEGG pathways of HFD group versus Ctrl group. (C) GO enrichment analysis of differentially expressed genes.

To clarify the molecular mechanisms of fucoidan treatment on dyslipidemia, we performed RNA‐seq on liver (Table S1). Heatmap data visualized the changes in total gene expression, clearly demonstrating the distribution of DEGs between CON, HFD, and HFD + FUC groups (Figure S2D). Volcano plot results showed that genes with higher expression levels of keratin family genes (Krt) in the HFD group compared to the CON group, including Krt15, Krt1, Krt5, Krt6a, and Krt14 (Figure 4A). While, defensin α 24 (Defa24) and Defa20, as well as the lipid transport protein, such as solute Carrier Family 5 Member 2 (Slc5a2) and Slc34a1 were downregulated in HFD group (Figure 4A). KEGG pathway enrichment analysis revealed that DEGs were enriched in multiple pathways, including NF‐κB signaling pathway, phosphatidylinositol signaling system, and IL‐17 signaling pathways (Figure 4B). Notably, environmental information processing pathways, such as lipid metabolic process, inflammatory response, and immune response, were altered (Figure 4C). These results indicate that an HFD disrupts the gene expression of inflammatory and immune pathways in liver tissue, thereby affecting its normal lipid metabolic functions.

**FIGURE 5:**
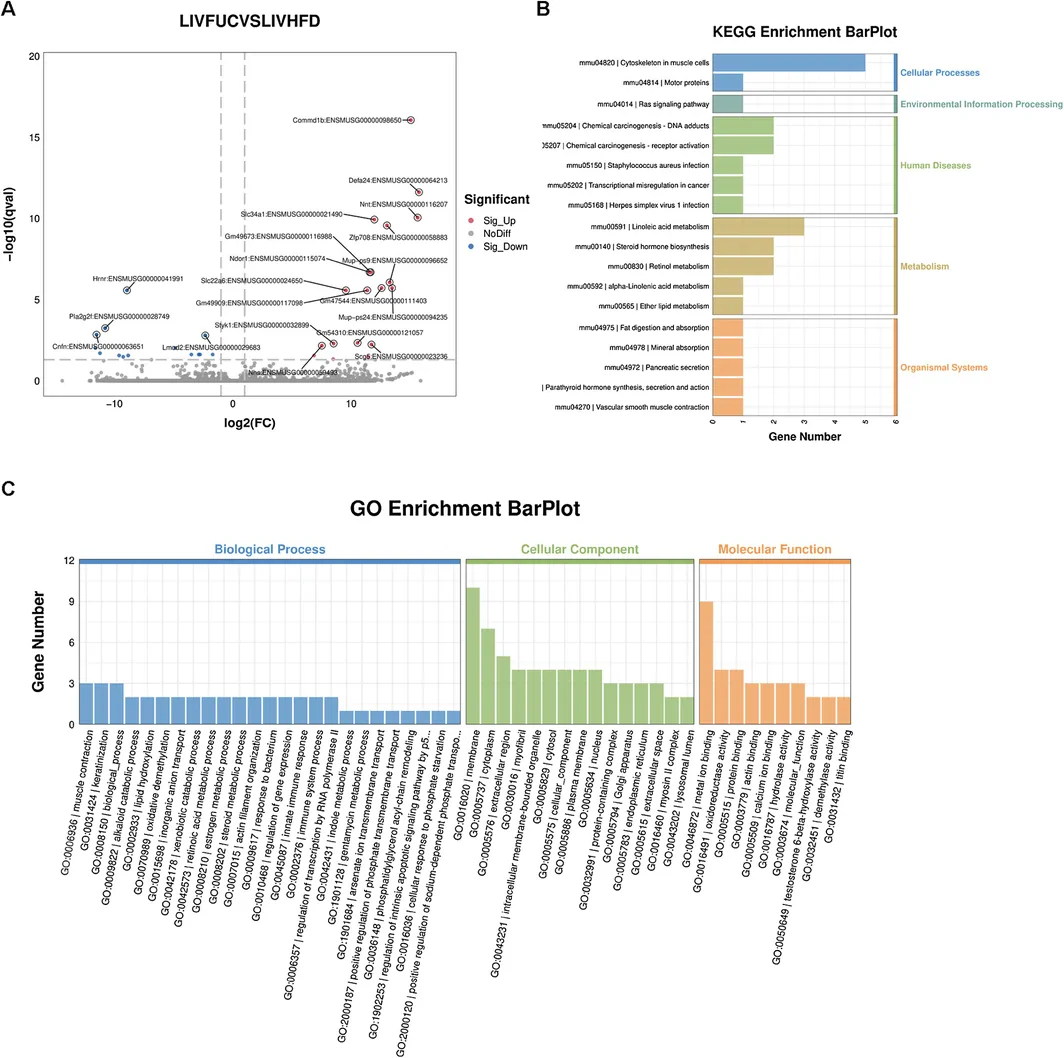
Fucoidan treatment regulates lipid metabolism to improve dyslipidemia. (A) Volcano plot of differentially genes in HFD + FUC group versus HFD group. Red dot represents upregulated gene, and blue dot represents downregulated gene. (B) The differentially genes enriched in KEGG pathways. (C) GO enrichment analysis of differentially genes.

**FIGURE 6:**
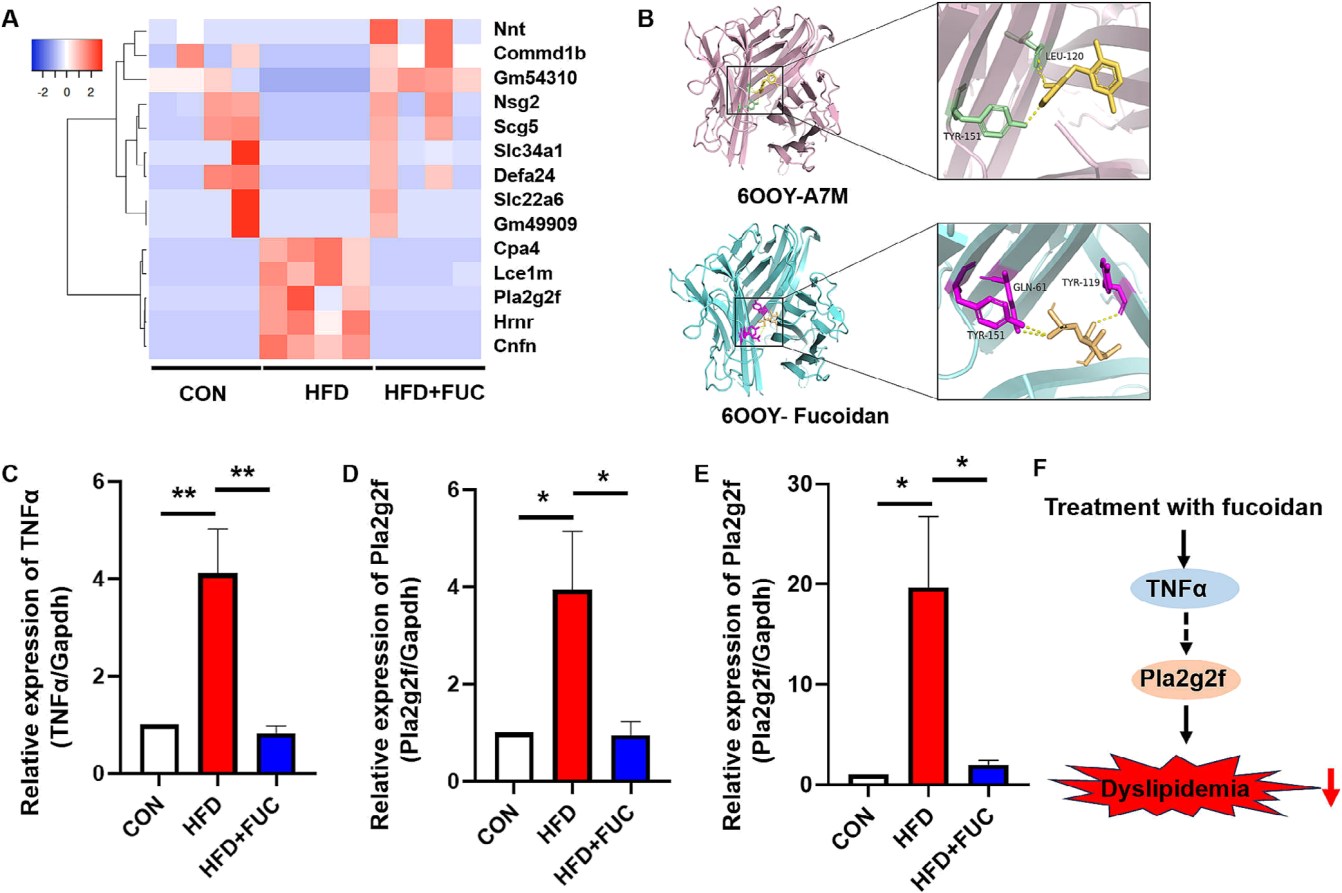
Fucoidan treatment regulates TNFα‐induced Pla2g2f expression, and thus improving dyslipidemia. (A) Heatmap result of differentially expressed genes altered in HFD group, and reversed by fucoidan treatment. (B) Molecular docking analysis in target protein TNFα and fucoidan. (C, D) The mRNA relative expression levels of TNFα (C) and Pla2g2f (D) in liver tissue (*n* = 4). (E) The mRNA relative expression levels of Pla2g2f mRNA expression in RAW264.7 cells (*n* = 6). (F) Molecular mechanisms of fucoidan against dyslipidemia. Arrows with a dashed line indicate the indirect relationship, arrows with a solid line indicate direct regulation or a direct connection. Data are expressed as mean ± SEM. *p < 0.05. **p < 0.01.

Additionally, the volcano plot shows the top 20 DEGs in the HFD + FUC group compared with the HFD group, including 16 upregulated genes and 4 downregulated genes (Figure 5A). Notably, Defa24, Slc34a1, nicotinamide nucleotide transhydrogenase (Nnt), and LincRNA Gm49909 were upregulated in the HFD + FUC group compared to the HFD group, which were downregulated DEGs in the HFD group compared to the CON group. KEGG pathway enrichment analysis showed that the DEGs were enriched in linoleic acid metabolism, fat digestion and absorption, and pancreatic secretion signaling pathways (Figure 5B). GO enrichment analysis also revealed altered lipid metabolic pathways involved in lipid hydroxylation, steroid metabolic process, and phosphatidylglycerol acyl‐chain remodeling pathways, as well as immune response associated pathways (Figure 5C). Furthermore, we examined DEGs altered by the HFD which could be reversed after fucoidan treatment (Figure 6A). A total of 14 DEGs were listed in the heatmap, and several genes associated with lipid metabolism were observed, such as copper metabolism domain containing 1 (Commd1b) and phospholipase A2 group IIF (Pla2g2f). Pla2g2f directly hydrolyzes membrane phospholipids to generate lysophospholipids and free fatty acids (Varastehpour et al. 2006), indicating that fucoidan treatment improves lipid metabolism dysregulation.

### 3.5 Fucoidan Treatment Improved Dyslipidemia by Regulating Pla2g2f Expression via TNFα‐Associated Signaling

Our previous network pharmacological result showed that the TNF pathway was crucial for fucoidan treatment against dyslipidemia. Molecular docking showed that fucoidan binds to TNFα with a binding energy of −6.7 kcal/mol, interacting with TYR‐119 (2.7 Å), TYR‐151 (3.1 Å), GLN‐61 (3.3 Å) sites (Figure 6B). Furthermore, qPCR results demonstrated a marked increase of TNFα in the HFD group compared to the CON group, whereas fucoidan treatment significantly decreased the TNFα expression level (Figure 5C). TNFα is a typical proinflammatory cytokine, which can induce a time‐dependent activation of Pla2g2 (Varastehpour et al. 2006). Pla2g2f belonged to the secreted phospholipases A2 (sPLA2), a crucial factor for efficient cholesterol absorption (Richmond et al. 2001). Our results showed that fucoidan treatment reversed the higher expression level of Pla2g2f induced by HFD (Figure 6D). To further investigate whether fucoidan regulates Pla2g2f expression through TNFα signaling, we found that 10 ng/mL TNFα stimulation significantly increased the mRNA expression level of Pla2g2f compared with the control group. Importantly, treatment with 50 μg/mL fucoidan markedly attenuated TNFα‐induced Pla2g2f expression. These findings provide evidence that fucoidan suppresses TNFα‐mediated Pla2g2f activation and further improve metabolic dysfunction (Figure 6F).

## 4 Discussion

In the present study, treatment with 50 mg/kg fucoidan did not attenuate HFD‐driven weight gain or alter food intake, particularly by reducing serum TC and hepatic cholesterol accumulation. Previous studies have reported that fucoidan administered at 100 mg/kg/day reduced serum TC and LDL‐C, and increased serum HDL‐C to improve lipid metabolic disorders in HFD‐fed mice (Huang et al. 2010; Park et al. 2016). Compared with these studies, our results demonstrate that a lower dose (50 mg/kg/day) is sufficient to improve cholesterol homeostasis, through reducing serum TC and normalizing HFD‐induced HDL‐C elevation, despite no significant change in LDL‐C. Notably, HFD‐induced elevation of HDL‐C has been reported to reflect increased circulating cholesterol rather than improved HDL function in mice (Podrini et al. 2013), suggesting that the reduction in serum HDL‐C following fucoidan treatment may represent normalization of serum cholesterol homeostasis. Consistent with the serum biochemical findings, fucoidan significantly reduced hepatic cholesterol accumulation and lipid droplet deposition, accompanied by decreased serum ALT and AST levels, indicating an improvement in HFD‐induced hepatic injury. Similar protective effects have also been reported in previous studies (Heeba and Morsy 2015; Yokota et al. 2016). Interestingly, hepatic RNA‐seq analysis revealed a trend toward restoration of Commd1 expression following fucoidan treatment. COMMD1 has been implicated in intracellular cholesterol trafficking and maintenance of hepatic cholesterol homeostasis through regulation of LDL receptor recycling (Bartuzi et al. 2016). Although serum LDL‐C remained unchanged, restoration of Commd1 expression supports that low‐dose fucoidan primarily regulates hepatic cholesterol metabolism.

Network pharmacology identified 116 targets, including TNF and AKT1, and these targets enriched in PI3K/AKT, TNF, and IL‐17 signaling pathways. GO analysis further suggested that fucoidan downregulates apoptosis and upregulates nitric‐oxide biosynthesis, supporting its anti‐inflammatory and anti‐oxidative stress roles. Accumulated studies have shown that fucoidan may be involved in regulating the PI3K/AKT pathway (Chu et al. 2025; Shiau et al. 2022). Activation of PI3K/AKT can enhance the activity of Nrf2 to enhance the antioxidant capacity and alleviate mitochondrial dysfunction (Cheng et al. 2023), and attribute to inflammatory cell recruitment and antioxidant responses (Lee, Koh, et al. 2022). Moreover, TNF and IL‐17 are proinflammatory cytokines, which were highly expressed in the hepatic injury model (Azam et al. 2018; Lee, Koh, et al. 2022). Among the identified hub genes, TNF was selected for further validation because it exhibited a high degree of connectivity in the PPI network and is widely recognized as a central inflammatory mediator linking obesity, chronic inflammation, and dyslipidemia. Accumulating evidence indicates that chronic inflammation is a critical contributor to dyslipidemia, and TNFα serves as a central mediator linking obesity‐induced inflammatory; notably, clinical studies have reported positive correlations between circulating TNFα levels and the severity of dyslipidemia, particularly hypercholesterolemia (Hong et al. 2022). Excess adiposity promotes the polarization and accumulation of pro‐inflammatory M1 macrophages within metabolic tissues, resulting in sustained production of TNFα and other inflammatory cytokines (Varra et al. 2024). Therefore, suppression of TNFα has been proposed as a promising strategy for improving obesity‐associated metabolic disorders.

RNA‐seq revealed that HFD altered hepatic expression of keratin genes (Krt family), defensins (Defa24, Defa20), and solute carriers (Slc5a2, Slc34a1). Krt is a defining property of the epithelium, involved in cell differentiation, as well as during acute or chronic inflammation and malignant transformation (Kalabusheva et al. 2023). Defensins and solute carriers are closely associated with hepatic steatosis (Maraga et al. 2023; Yu et al. 2023), reflecting HFD‐induced disrupted barrier and transport functions. Notably, fucoidan treatment reversed the HFD‐induced downregulation of Slc34a1, Defa24, and Nnt. Slc34a1 encodes a sodium‐phosphate cotransporter that is essential for intestinal inorganic phosphate transportation and metabolic homeostasis (Costa et al. 2023). Defa24 belongs to the α‐defensin that maintains the epithelial barrier; both play essential roles in preserving intestinal epithelial integrity and function, (Levi et al. 2019). Restoration of these genes may contribute to improve gut barrier function and gut–liver axis communication under HFD conditions. In addition, Nnt generates mitochondrial NADPH to support glucose‐stimulated insulin secretion, and its deficiency has been associated with increased susceptibility to diet‐induced metabolic disorders (Nicholson et al. 2010). Collectively, these findings suggest that fucoidan may be partly mediated dyslipidemia through restoration of Defa24, Slc34a1, and Nnt expression, thereby improving gut–liver axis homeostasis, mitochondrial function, and lipid metabolic balance. However, the functional roles of these genes in mediating the lipid metabolic regulation of fucoidan remain speculative and require further experimental validation.

In the present study, network pharmacology identified TNF as one of the core targets potentially associated with the lipid metabolic regulation of fucoidan, and molecular docking suggested a potential interaction between fucoidan and TNFα. Moreover, a previous study reported that TNFα can stimulate secretory phospholipase A2 (sPLA2) activity through transcriptional regulation of PLA2 family members (Oka and Arita 1991). Among these enzymes, Pla2g2f belongs to the sPLA2 family and catalyzes the hydrolysis of membrane phospholipids, generating free fatty acids and lysophospholipids that participate in inflammatory amplification and lipid metabolic remodeling (Fonteh et al. 1998; Six and Dennis 2000). Notably, our RNA‐seq analysis identified Pla2g2f as one of the key genes reversed by fucoidan treatment, and qPCR further confirmed that fucoidan significantly suppressed HFD‐induced Pla2g2f expression in liver tissues. Furthermore, fucoidan significantly attenuated TNFα‐induced Pla2g2f expression in TNFα‐stimulated RAW264.7 macrophages, suggesting that fucoidan modulates downstream inflammatory responses triggered by TNFα. Given the central role of macrophage‐derived TNFα in obesity‐associated inflammation, these findings suggest that regulation of TNFα‐associated inflammatory signaling and subsequent suppression of Pla2g2f expression may contribute to the protective effect of fucoidan against HFD‐induced dyslipidemia. Collectively, our findings demonstrate that fucoidan exerts beneficial effects against HFD‐induced dyslipidemia through multiple complementary mechanisms involving cholesterol metabolism, and attenuation of TNF‐associated inflammatory responses, highlighting its potential as a functional food ingredient for dyslipidemia prevention and management.

## Author Contributions

**Jicai Lai:** conceptualization, formal analysis, project administration, supervision, writing – review and editing. **Yuting Zeng:** data curation. **Ruting Zheng:** resources, investigation. **Daohuang Luo:** investigation, visualization. **Lizhi Peng:** conceptualization, investigation, writing – original draft, validation. **Qiaoling Kong:** supervision, project administration, formal analysis. **Ziqiong Liao:** formal analysis, investigation. **Jiaqi Liu:** writing – review and editing, supervision. **Ruiming Li:** data curation, funding acquisition, writing – original draft.

## Funding

This study was partly granted by the Open Fund of Shenzhen Key Laboratory of Chinese Medicine Active Substance Screening and Translational Research (ZDSYS20220606100801003).

## Conflicts of Interest

The authors declare no conflicts of interest.

## Supporting information


**Figure S1:** Characterization of fucoidan.
A. HPGPC chromatogram of fucoidan showing a major symmetrical peak at approximately 10.61 min, indicating a relatively homogeneous molecular weight distribution. B. HPLC chromatogram of PMP‐derivatized monosaccharides following acid hydrolysis of fucoidan.

**Figure S2:** Low‐dose of fucoidan treatment alters gene expression in liver tissue. A–C. The weights of adipose tissue, including inguinal white adipose tissue (iWAT) (A), epididymal white adipose tissue (eWAT) (B) and brown adipose tissue (BAT) (C) in CON, HFD and HFD + FUC groups. D. Heatmap of total genes in transcriptomics data. Results presented as means ± SEM of five independent experiments (*n* = 5). *p < 0.05. **p < 0.01.

**Table S1:** Differentially expressed genes (DEGs) in the liver of HFD‐fed mice following fucoidan treatment. RNA‐seq was performed on liver tissues from four biological replicates per group (*n* = 4). DEGs were identified using DESeq2 with the thresholds of |log_2_(fold change)| ≥ 1 and adjusted *p*‐value < 0.05. Shown are the gene symbol, log_2_ fold change, adjusted *p*‐value.

**Table S2:** Physicochemical characterization of fucoidan.
Monosaccharide composition, total sugar content and sulfate content of fucoidan.

## Data Availability

The data that support the findings of this study are available from the corresponding author upon reasonable request.

## References

[fsn372159-bib-0001] Atashrazm, F. , R. M. Lowenthal , G. M. Woods , A. F. Holloway , and J. L. Dickinson . 2015. “Fucoidan and Cancer: A Multifunctional Molecule With Anti‐Tumor Potential.” Marine Drugs 13: 2327–2346. 25874926 10.3390/md13042327 PMC4413214

[fsn372159-bib-0002] Azam, F. , N. Sheikh , G. Ali , and A. Tayyeb . 2018. “Fagonia Indica Repairs Hepatic Damage Through Expression Regulation of Toll‐Like Receptors in a Liver Injury Model.” Journal of Immunology Research 2018: 7967135. 30057922 10.1155/2018/7967135 PMC6051044

[fsn372159-bib-0003] Bartuzi, P. , D. D. Billadeau , R. Favier , et al. 2016. “CCC‐ and WASH‐Mediated Endosomal Sorting of LDLR Is Required for Normal Clearance of Circulating LDL.” Nature Communications 7: 10961. 10.1038/ncomms10961 PMC4792963 26965651

[fsn372159-bib-0004] Boo, H. J. , J. Y. Hong , S. C. Kim , et al. 2013. “The Anticancer Effect of Fucoidan in PC‐3 Prostate Cancer Cells.” Marine Drugs 11: 2982–2999. 23966032 10.3390/md11082982 PMC3766877

[fsn372159-bib-0005] Carr, R. M. , and R. S. Ahima . 2016. “Pathophysiology of Lipid Droplet Proteins in Liver Diseases.” Experimental Cell Research 340: 187–192. 26515554 10.1016/j.yexcr.2015.10.021 PMC4744586

[fsn372159-bib-0006] Chapman, M. J. , J. S. Redfern , M. E. Mcgovern , and P. Giral . 2010. “Niacin and Fibrates in Atherogenic Dyslipidemia: Pharmacotherapy to Reduce Cardiovascular Risk.” Pharmacology & Therapeutics 126: 314–345. 20153365 10.1016/j.pharmthera.2010.01.008

[fsn372159-bib-0007] Chehade, J. M. , M. Gladysz , and A. D. Mooradian . 2013. “Dyslipidemia in Type 2 Diabetes: Prevalence, Pathophysiology, and Management.” Drugs 73: 327–339. 23479408 10.1007/s40265-013-0023-5

[fsn372159-bib-0008] Cheng, Y. , Y. Gao , J. Li , et al. 2023. “TrkB Agonist N‐Acetyl Serotonin Promotes Functional Recovery After Traumatic Brain Injury by Suppressing Ferroptosis via the PI3K/Akt/Nrf2/Ferritin H Pathway.” Free Radical Biology & Medicine 194: 184–198. 36493983 10.1016/j.freeradbiomed.2022.12.002

[fsn372159-bib-0009] Chu, X. , X. Wang , K. Feng , Y. Bi , Y. Xin , and S. Liu . 2025. “Fucoidan Ameliorates Lipid Accumulation, Oxidative Stress, and NF‐kappaB‐Mediated Inflammation by Regulating the PI3K/AKT/Nrf2 Signaling Pathway in a Free Fatty Acid‐Induced NAFLD Spheroid Model.” Lipids in Health and Disease 24: 55. 39962463 10.1186/s12944-025-02483-z PMC11831825

[fsn372159-bib-0010] Costa, A. V. D. , I. C. Rattes , C. P. Goes , L. H. G. Lobo , L. B. E. Barreto , and P. Gama . 2023. “Breastfeeding Lifespan Control of Growth, Maintenance, and Metabolism of Small Intestinal Epithelium.” Journal of Cellular Physiology 238: 2304–2315. 37555566 10.1002/jcp.31089

[fsn372159-bib-0011] Fitton, H. J. , D. S. Stringer , A. Y. Park , and S. N. Karpiniec . 2019. “Therapies From Fucoidan: New Developments.” Marine Drugs 17: 571. 31601041 10.3390/md17100571 PMC6836154

[fsn372159-bib-0012] Fonteh, A. N. , J. M. Samet , M. Surette , W. Reed , and F. H. Chilton . 1998. “Mechanisms That Account for the Selective Release of Arachidonic Acid From Intact Cells by Secretory Phospholipase A.” Biochimica et Biophysica Acta (BBA) ‐ Lipids and Lipid Metabolism 1393: 253–266. 9748613 10.1016/s0005-2760(98)00079-4

[fsn372159-bib-0013] Heeba, G. H. , and M. A. Morsy . 2015. “Fucoidan Ameliorates Steatohepatitis and Insulin Resistance by Suppressing Oxidative Stress and Inflammatory Cytokines in Experimental Non‐Alcoholic Fatty Liver Disease.” Environmental Toxicology and Pharmacology 40: 907–914. 26498267 10.1016/j.etap.2015.10.003

[fsn372159-bib-0014] Hong, N. , Y. Lin , Z. Ye , et al. 2022. “The Relationship Between Dyslipidemia and Inflammation Among Adults in East Coast China: A Cross‐Sectional Study.” Frontiers in Immunology 13: 937201. 36032093 10.3389/fimmu.2022.937201 PMC9403313

[fsn372159-bib-0015] Hu, T. , H. Wu , Y. Liao , et al. 2026. “Structural Characterization and Anti‐Lung Cancer Activity of Fucoidans Derived From Saccharina Japonica.” International Journal of Biological Macromolecules 374: 153306. 42392389 10.1016/j.ijbiomac.2026.153306

[fsn372159-bib-0016] Huang, J. , J. Huang , Y. Li , et al. 2021. “Fucoidan Protects Against High‐Fat Diet‐Induced Obesity and Modulates Gut Microbiota in Institute of Cancer Research Mice.” Journal of Medicinal Food 24: 1058–1067. 10.1089/jmf.2021.K.0030 34668763

[fsn372159-bib-0017] Huang, L. , K. Wen , X. Gao , and Y. Liu . 2010. “Hypolipidemic Effect of Fucoidan From Laminaria Japonica in Hyperlipidemic Rats.” Pharmaceutical Biology 48: 422–426. 20645721 10.3109/13880200903150435

[fsn372159-bib-0018] Kalabusheva, E. P. , A. S. Shtompel , A. L. Rippa , S. V. Ulianov , S. V. Razin , and E. A. Vorotelyak . 2023. “A Kaleidoscope of Keratin Gene Expression and the Mosaic of Its Regulatory Mechanisms.” International Journal of Molecular Sciences 24: 5603. 36982676 10.3390/ijms24065603 PMC10052683

[fsn372159-bib-0019] Katsiki, N. , D. P. Mikhailidis , and C. S. Mantzoros . 2016. “Non‐Alcoholic Fatty Liver Disease and Dyslipidemia: An Update.” Metabolism 65: 1109–1123. 27237577 10.1016/j.metabol.2016.05.003

[fsn372159-bib-0020] Kim, M. J. , J. Jeon , and J. S. Lee . 2014. “Fucoidan Prevents High‐Fat Diet‐Induced Obesity in Animals by Suppression of Fat Accumulation.” Phytotherapy Research 28: 137–143. 23580241 10.1002/ptr.4965

[fsn372159-bib-0021] Lee, H. G. , T. U. Jayawardena , K. M. Song , Y. S. Choi , Y. J. Jeon , and M. C. Kang . 2022. “Dietary Fucoidan From a Brown Marine Algae ( ) Attenuates Lipid Accumulation in Differentiated 3T3‐L1 Cells and Alleviates High‐Fat Diet‐Induced Obesity in Mice.” Food and Chemical Toxicology 162: 112862. 35157925 10.1016/j.fct.2022.112862

[fsn372159-bib-0022] Lee, S. M. , D. H. Koh , D. W. Jun , et al. 2022. “Auranofin Attenuates Hepatic Steatosis and Fibrosis in Nonalcoholic Fatty Liver Disease via NRF2 and NF‐ kappaB Signaling Pathways.” Clinical and Molecular Hepatology 28: 827–840. 35730208 10.3350/cmh.2022.0068 PMC9597229

[fsn372159-bib-0023] Levi, M. , E. Gratton , I. C. Forster , et al. 2019. “Mechanisms of Phosphate Transport.” Nature Reviews. Nephrology 15: 482–500. 31168066 10.1038/s41581-019-0159-y

[fsn372159-bib-0024] Li, J. K. , X. W. Wan , Y. H. Li , et al. 2025. “Anti‐Obesity Functions of Fucoidan Conducted by Bioinformatics and Validation Findings Targeting of Autophagy.” Carbohydrate Polymer Technologies and Applications 9: 100609.

[fsn372159-bib-0025] Lin, Q. , L. Zheng , W. Pan , et al. 2025. “Fucoidan Derived From Saccharina Japonica: Structural Characterization and Amelioration of High‐Fat‐Diet Induced Obesity in Male C57BL/6 J Mice via Modulating the Gut Microbiota and Lipid Metabolites.” International Journal of Biological Macromolecules 310: 143026. 40216141 10.1016/j.ijbiomac.2025.143026

[fsn372159-bib-0026] Liu, J. , J. Li , W. Chen , et al. 2021. “Comprehensive Evaluation of the Metabolic Effects of Porcine CRTC3 Overexpression on Subcutaneous Adipocytes With Metabolomic and Transcriptomic Analyses.” Journal of Animal Science and Biotechnology 12: 19. 33653408 10.1186/s40104-021-00546-6 PMC7927250

[fsn372159-bib-0027] Maraga, E. , R. Safadi , J. Amer , A. A. Higazi , and R. A. Fanne . 2023. “Alleviation of Hepatic Steatosis by Alpha‐Defensin Is Associated With Enhanced Lipolysis.” Medicina 59: 983. 37241215 10.3390/medicina59050983 PMC10220962

[fsn372159-bib-0028] Modica, R. , A. LA Salvia , A. Liccardi , et al. 2024. “Dyslipidemia, Lipid‐Lowering Agents and Neuroendocrine Neoplasms: New Horizons.” Endocrine 85: 520–531. 38509261 10.1007/s12020-024-03767-7 PMC11291585

[fsn372159-bib-0029] Nicholson, A. , P. C. Reifsnyder , R. D. Malcolm , et al. 2010. “Diet‐Induced Obesity in Two C57BL/6 Substrains With Intact or Mutant Nicotinamide Nucleotide Transhydrogenase (Nnt) Gene.” Obesity (Silver Spring) 18: 1902–1905. 20057372 10.1038/oby.2009.477 PMC2888716

[fsn372159-bib-0030] Nussbaumerova, B. , and H. Rosolova . 2023. “Obesity and Dyslipidemia.” Current Atherosclerosis Reports 25: 947–955. 37979064 10.1007/s11883-023-01167-2

[fsn372159-bib-0031] Oka, S. , and H. Arita . 1991. “Inflammatory Factors Stimulate Expression of Group II Phospholipase A2 in Rat Cultured Astrocytes. Two Distinct Pathways of the Gene Expression.” Journal of Biological Chemistry 266: 9956–9960. 2033082

[fsn372159-bib-0032] Park, J. , M. Yeom , and D. H. Hahm . 2016. “Fucoidan Improves Serum Lipid Levels and Atherosclerosis Through Hepatic SREBP‐2‐Mediated Regulation.” Journal of Pharmacological Sciences 131: 84–92. 27094367 10.1016/j.jphs.2016.03.007

[fsn372159-bib-0033] Peng, L. , R. Li , W. Xie , et al. 2025. “Fucoidan Reinforce Mitophagy in Brown Adipose Tissue Thermogenesis to Protect Against Obesity.” Carbohydrate Polymer Technologies and Applications 12: 101003.

[fsn372159-bib-0034] Podrini, C. , E. L. Cambridge , C. J. Lelliott , et al. 2013. “High‐Fat Feeding Rapidly Induces Obesity and Lipid Derangements in C57BL/6N Mice.” Mammalian Genome 24: 240–251. 23712496 10.1007/s00335-013-9456-0 PMC3685703

[fsn372159-bib-0035] Richmond, B. L. , A. C. Boileau , S. Zheng , et al. 2001. “Compensatory Phospholipid Digestion Is Required for Cholesterol Absorption in Pancreatic Phospholipase A(2)‐Deficient Mice.” Gastroenterology 120: 1193–1202. 11266383 10.1053/gast.2001.23254

[fsn372159-bib-0036] Shiau, J. P. , Y. T. Chuang , Y. B. Cheng , et al. 2022. “Impacts of Oxidative Stress and PI3K/AKT/mTOR on Metabolism and the Future Direction of Investigating Fucoidan‐Modulated Metabolism.” Antioxidants 11: 911. 35624775 10.3390/antiox11050911 PMC9137824

[fsn372159-bib-0037] Six, D. A. , and E. A. Dennis . 2000. “The Expanding Superfamily of Phospholipase A(2) Enzymes: Classification and Characterization.” Biochimica et Biophysica Acta 1488: 1–19. 11080672 10.1016/s1388-1981(00)00105-0

[fsn372159-bib-0038] Varastehpour, A. , T. Radaelli , J. Minium , et al. 2006. “Activation of Phospholipase A2 Is Associated With Generation of Placental Lipid Signals and Fetal Obesity.” Journal of Clinical Endocrinology and Metabolism 91: 248–255. 16249288 10.1210/jc.2005-0873

[fsn372159-bib-0039] Varra, F. N. , M. Varras , V. K. Varra , and P. Theodosis‐Nobelos . 2024. “Molecular and Pathophysiological Relationship Between Obesity and Chronic Inflammation in the Manifestation of Metabolic Dysfunctions and Their Inflammation‐Mediating Treatment Options (Review).” Molecular Medicine Reports 29: 95. 38606791 10.3892/mmr.2024.13219 PMC11025031

[fsn372159-bib-0040] Vekic, J. , A. Stefanovic , and A. Zeljkovic . 2023. “Obesity and Dyslipidemia: A Review of Current Evidence.” Current Obesity Reports 12: 207–222. 37410248 10.1007/s13679-023-00518-z

[fsn372159-bib-0041] Yokota, T. , K. Nomura , M. Nagashima , and N. Kamimura . 2016. “Fucoidan Alleviates High‐Fat Diet‐Induced Dyslipidemia and Atherosclerosis in ApoE(Shl) Mice Deficient in Apolipoprotein E Expression.” Journal of Nutritional Biochemistry 32: 46–54. 27142736 10.1016/j.jnutbio.2016.01.011

[fsn372159-bib-0042] Yu, H. , H. Tang , M. Wang , et al. 2023. “Effects of Total Flavones of *Abelmoschus manihot* (L.) on the Treatment of Diabetic Nephropathy via the Activation of Solute Carriers in Renal Tubular Epithelial Cells.” Biomedicine & Pharmacotherapy 169: 115899. 37984306 10.1016/j.biopha.2023.115899

[fsn372159-bib-0043] Zeljkovic, A. , J. Vekic , and A. Stefanovic . 2024. “Obesity and Dyslipidemia in Early Life: Impact on Cardiometabolic Risk.” Metabolism 156: 155919. 38653373 10.1016/j.metabol.2024.155919

[fsn372159-bib-0044] Zheng, Y. , T. Liu , Z. Wang , Y. Xu , Q. Zhang , and D. Luo . 2018. “Low Molecular Weight Fucoidan Attenuates Liver Injury via SIRT1/AMPK/PGC1alpha Axis in Db/Db Mice.” International Journal of Biological Macromolecules 112: 929–936. 29447962 10.1016/j.ijbiomac.2018.02.072

[fsn372159-bib-0045] Zuo, J. H. , Y. Zhang , Y. Wu , et al. 2022. “Fucoidan Ameliorates Diet‐Induced Obesity Through Enhancing Thermogenesis of Adipose Tissues and Modulating Gut Microbiota.” International Journal of Biological Macromolecules 216: 728–740. 35907465 10.1016/j.ijbiomac.2022.07.184

